# Supplementary material for: Artificial Intelligence in Digital Pathology for Bladder Cancer: Hype or Hope? A Systematic Review
Source: Cancers (Basel). 2023 Sep 12;15(18):4518. doi: 10.3390/cancers15184518 (PMC10526515; doi:10.3390/cancers15184518)
Supplement: Supplementary file 1 [file cancers-15-04518-s001.zip › cancers-2554781-supplementary.pdf]

cancers

MDPI

Supplementary Table S1: Search terms and results on March 2022

Database searched	Platform	Years of coverage	Records	Records after duplicates remove
Embase	Embase.com	1971 - Present	794	767
Medline ALL	Ovid	1946 - Present	865	552
Web of Science Core Collection*	Web of Knowledge	1975 - Present	498	171
Cochrane Central Register of Controlled Trials	Wiley	1992 - Present	28	20
Additional Search Engines: Google Scholar			100	55
	**Total**		**2285**	**1565**

*Science Citation Index Expanded (1975-present); Social Sciences Citation Index (1975-present); Arts & Humanities Citation Index (1975-present); Conference Proceedings Citation Index- Science (1990-present); Conference Proceedings Citation Index- Social Science & Humanities (1990-present); Emerging Sources Citation Index (2005-present)

Embase

('bladder cancer'/exp OR 'transitional cell carcinoma'/de OR 'bladder tumor'/de OR 'bladder cancer cell line'/exp OR 'bladder carcinogenesis'/de OR 'bladder cancer detection kit'/de OR (((bladder* OR urothelial*) NEAR/3 (cancer* OR tumor* OR tumour* OR malign* OR neoplas* OR carcino* OR metastas*)) OR MIBC OR variant-histolog*):ab,ti,kw) AND ('digital pathology'/de OR 'machine learning'/de OR 'artificial intelligence'/exp OR 'computer assisted diagnosis'/de OR 'deep learning'/de OR 'fuzzy logic'/de OR 'fuzzy system'/de OR 'artificial neural network'/exp OR 'random forest'/de OR 'supervised learning'/de OR 'unsupervised machine learning'/de OR 'segmentation algorithm'/exp OR (((digital*) NEAR/3 (patholog*)) OR ((machine* OR deep OR supervised OR unsupervised) NEAR/3 (learning*)) OR ((artificial* OR machine*) NEAR/3 (intelligence*)) OR ((neural*) NEAR/3 (network*)) OR ((computer) NEAR/3 (diagnos* OR interpretat* OR recogni*)) OR ((fuzzy) NEAR/3 (logic* OR model* OR system*)) OR ((random*) NEAR/3 (forest*)) OR ((stain* OR colour* OR color*) NEAR/3 (normali* OR standardi*)) OR ((segmentation*) NEAR/3 (algor* OR imag*)) OR explainable-AI*):ab,ti,kw)

Medline

(exp Urinary Bladder Neoplasms/ OR Carcinoma, Transitional Cell/ OR (((bladder* OR urothelial*) ADJ3 (cancer* OR tumor* OR tumour* OR malign* OR neoplas* OR carcino* OR metastas*)) OR MIBC OR variant-histolog*).ab,ti,kf.) AND (exp Artificial Intelligence/ OR Diagnosis, Computer-Assisted/ OR Fuzzy Logic/ OR exp Neural Networks, Computer/ OR (((digital*) ADJ3 (patholog*)) OR ((machine* OR deep OR supervised OR unsupervised) ADJ3 (learning*)) OR ((artificial* OR machine*) ADJ3 (intelligence*)) OR ((neural*) ADJ3 (network*)) OR ((computer) ADJ3 (diagnos* OR interpretat* OR recogni*)) OR ((fuzzy) ADJ3 (logic* OR model* OR system*)) OR ((random*) ADJ3 (forest*)) OR ((stain* OR colour* OR color*) ADJ3 (normali* OR standardi*)) OR ((segmentation*) ADJ3 (algor* OR imag*)) OR explainable-AI*).ab,ti,kf.)

Cochrane

((((bladder* OR urothelial*) NEAR/3 (cancer* OR tumor* OR tumour* OR malign* OR neoplas* OR carcino* OR metastas*)) OR MIBC OR variant NEXT histolog*):ab,ti,kw) AND ((((digital*) NEAR/3 (patholog*)) OR ((machine* OR deep OR supervised OR unsupervised) NEAR/3 (learning*)) OR ((artificial* OR machine*) NEAR/3 (intelligence*)) OR

((neural*) NEAR/3 (network*)) OR ((computer) NEAR/3 (diagnos* OR interpretat* OR recogni*)) OR ((fuzzy) NEAR/3 (logic* OR model* OR system*)) OR ((random*) NEAR/3 (forest*)) OR ((stain* OR colour* OR color*) NEAR/3 (normali* OR standardi*)) OR ((segmentation*) NEAR/3 (algor* OR imag*)) OR explainable NEXT AI*):ab,ti,kw)

Web of Science

TS=(((((bladder* OR urothelial*) NEAR/2 (cancer* OR tumor* OR tumour* OR malign* OR neoplas* OR carcino* OR metastas*)) OR MIBC OR variant-histolog*)) AND ((((digital*) NEAR/2 (patholog*)) OR ((machine* OR deep OR supervised OR unsupervised) NEAR/2 (learning*)) OR ((artificial* OR machine*) NEAR/2 (intelligence*)) OR ((neural*) NEAR/2 (network*)) OR ((computer) NEAR/2 (diagnos* OR interpretat* OR recogni*)) OR ((fuzzy) NEAR/2 (logic* OR model* OR system*)) OR ((random*) NEAR/2 (forest*)) OR ((stain* OR colour* OR color*) NEAR/2 (normali* OR standardi*)) OR ((segmentation*) NEAR/2 (algor* OR imag*)) OR explainable-AI*)))

Google Scholar

'bladder cancer|tumor|tumour|neoplasm|carcinoma|metastasis'|MIBC 'digital pathology'|AI|'machine|deep|supervised|unsupervised learning'|'artificial|machine intelligence'|'neural network'|fuzzy|'computer diagnosis|interpretation|recognition'
